# Zipf's Law Leads to Heaps' Law: Analyzing Their Relation in Finite-Size Systems

**DOI:** 10.1371/journal.pone.0014139

**Published:** 2010-12-02

**Authors:** Linyuan Lü, Zi-Ke Zhang, Tao Zhou

**Affiliations:** 1 Web Sciences Center, University of Electronic Science and Technology of China, Chengdu, People's Republic of China; 2 Department of Physics, University of Fribourg, Fribourg, Switzerland; 3 Department of Modern Physics, University of Science and Technology of China, Hefei, People's Republic of China; Indiana University, United States of America

## Abstract

**Background:**

Zipf's law and Heaps' law are observed in disparate complex systems. Of particular interests, these two laws often appear together. Many theoretical models and analyses are performed to understand their co-occurrence in real systems, but it still lacks a clear picture about their relation.

**Methodology/Principal Findings:**

We show that the Heaps' law can be considered as a derivative phenomenon if the system obeys the Zipf's law. Furthermore, we refine the known approximate solution of the Heaps' exponent provided the Zipf's exponent. We show that the approximate solution is indeed an asymptotic solution for infinite systems, while in the finite-size system the Heaps' exponent is sensitive to the system size. Extensive empirical analysis on tens of disparate systems demonstrates that our refined results can better capture the relation between the Zipf's and Heaps' exponents.

**Conclusions/Significance:**

The present analysis provides a clear picture about the relation between the Zipf's law and Heaps' law without the help of any specific stochastic model, namely the Heaps' law is indeed a derivative phenomenon from the Zipf's law. The presented numerical method gives considerably better estimation of the Heaps' exponent given the Zipf's exponent and the system size. Our analysis provides some insights and implications of real complex systems. For example, one can naturally obtained a better explanation of the accelerated growth of scale-free networks.

## Introduction

Giant strides in Complexity Sciences have been the direct outcome of efforts to uncover the universal laws that govern disparate systems. Zipf's law [1] and Heaps' law [2] are two representative examples. In 1940s, Zipf found a certain scaling law in the distribution of the word frequencies. Ranking all the words in descending order of occurrence frequency and denoting by 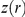 the frequency of the word with rank 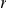, the Zipf's law reads 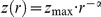, where 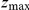 is the maximal frequency and 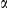 is the so-called Zipf's exponent. This power-law frequency-rank relation indicates a power-law probability distribution of the frequency itself, say 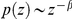 with 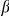 equal to 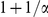 (see **Materials and Methods**). As a signature of complex systems, the Zipf's law is observed everywhere [3]: these include the distributions of firm sizes [4], wealths and incomes [5], paper citations [6], gene expressions [7], sizes of blackouts [8], family names [9], city sizes [10], personal donations [11], chess openings [12], traffic loads caused by YouTube videos [13], and so on. Accordingly, many mechanisms are put forward to explain the emergence of the Zipf's law [14], [15], such as the *rich gets richer* [16], [17], the *self-organized criticality* [18], *Markov Processes* [19], *aggregation of interacting individuals* [20], *optimization designs* [21] and the *least effort principle* [22]. To name just a few.

Heaps' law [2] can also be applied in characterizing natural language processing, according to which the vocabulary size grows in a sublinear function with document size, say 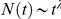 with 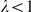, where 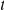 denotes the total number of words and 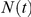 is the number of distinct words. One ingredient causing such a sublinear growth may be the memory and bursty nature of human language [23]–[25]. A particular interesting phenomenon is the coexistence of the Zipf's law and Heaps' law. Gelbukh and Sidorov [26] observed these two laws in English, Russian and Spanish texts, with different exponents depending on languages. Similar results were recently reported for the corpus of web texts [27], including the *Industry Sector database*, the *Open Directory* and the *English Wikipedia*. Besides the statistical regularities of text, the occurrences of tags for online resources [28], [29], keywords for scientific publications [30], words contained by web pages resulted from web searching [31], and identifiers in modern Java, C++ and C programs [32] also simultaneously display the Zipf's law and Heaps' law. Benz *et al.* [33] reported the Zipf's law of the distribution of the features of small organic molecules, together with the Heaps' law about the number of unique features. In particular, the Zipf's law and Heaps' law are closely related to the evolving networks. It is well-known that some networks grow in an accelerating manner [34], [35] and have scale-free structures (see for example the WWW [36] and Internet [37]), in fact, the former property corresponds to the Heaps' law that the number of nodes grows in a sublinear form with the total degree of nodes, while the latter is equivalent to the Zipf's law for degree distribution.

Baeza-Yates and Navarro [38] showed that the two laws are related: when 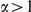, it can be derived that if both the Zipf's law and Heaps' law hold, 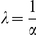. By using a more sophisticated approach, Leijenhorst and Weide [39] generalized this result from the Zipf's law to the Mandelbrot's law [40] where 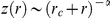 and 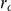 is a constant. Based on a variant of the Simon model [16], Montemurro and Zanette [41], [42] showed that the Zipf's law is a result from the Heaps' law with 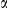 depending on 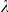 and the modeling parameter. Also based on a stochastic model, Serrano *et al.* [27] claimed that the Zipf's law can result in the Heaps' law when 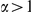, and the Heaps' exponent is 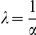. In this paper, we prove that for an evolving system with a stable Zipf's exponent, the Heaps' law can be directly derived from the Zipf's law without the help of any specific stochastic model. The relation 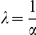 is only an asymptotic solution hold for very-large-size systems with 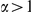. We will refine this result for finite-size systems with 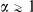 and complement it with 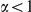. In particular, we analyze the effects of system size on the Heaps' exponent, which are completely ignored in the literature. Extensive empirical analysis on tens of disparate systems ranging from keyword occurrences in scientific journals to spreading patterns of the novel virus influenza A (H1N1) has demonstrated that the refined results presented here can better capture the relation between Zipf's and Heaps' exponents. In particular, our results agree well with the evolving regularities of the accelerating networks and suggest that the accelerating growth is necessary to keep a stable power-law degree distribution. Whereas the majority of studies on the Heaps' law are limited in linguistics, our work opens up the door to a much wider horizon that includes many complex systems.

## Results

### Analytical Results

For simplicity of depiction, we use the language of word statistics in text, where 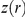 denotes the frequency of the word with rank 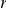. However, the results are not limited to language systems. Note that 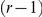 is the very number of distinct words with frequency larger than 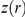. Denoting by 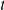 the total number of word occurrences (i.e., size of the text) and 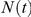 the corresponding number of distinct words, then
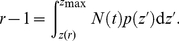
(1)Note that 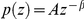 with 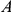 a constant. According to the normalization condition 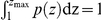, when 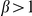 and 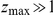 (these two conditions are hold for most real systems), 
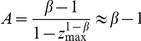
. Substituting 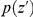 in Eq. 1 by 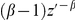, we have
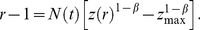
(2)According to the Zipf's law 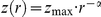 and the relation between the Zipf's and power-law exponents 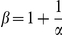, the right part of Eq. 2 can be expressed in term of 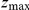 and 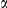, as
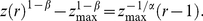
(3)Combine Eq. 1 and Eq. 3, we can obtain the estimation of 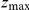, as
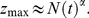
(4)Obviously, the text size 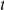 is the sum of all words' occurrences, say
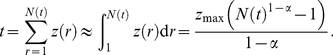
(5)Notice that the summation 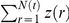 is larger than the integration 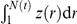. The relative error of this approximation, for 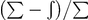, increases with the increasing of 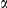 and decreases with the increasing of 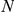 (see Figure S1 the numerical results on the sensitivity of relative errors to parameters 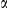 and 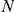). Substituting 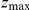 by Eq. 4, it arrives to the relation between 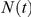 and 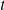:
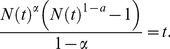
(6)The direct comparison between the empirical observation and Eq. 6, as well as an improved version of Eq. 6, is shown in **Materials and Methods**. Clearly, Eq. 6 is not a simply power-law form as described by the Heaps' law. We will see that the Heaps' law is an approximate result that can be derived from Eq. 6. Actually, when 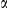 is considerably larger than 1, 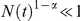 and 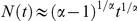; while if 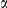 is considerably smaller than 1, 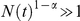 and 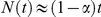. This approximated result can be summarized as
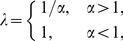
(7)which is in accordance with the previous analytical results [29], [38], [39] for 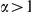 and has complemented the case for 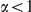.

**Figure 1:**
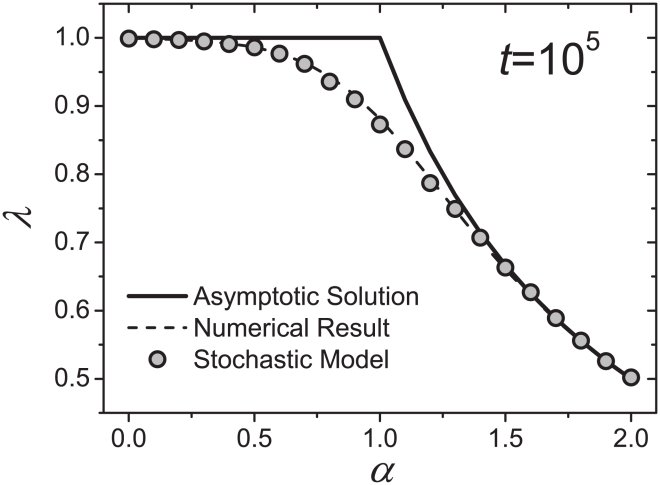
Relationship between the Heaps' exponent 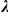 and Zipf's exponent 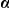. The solid curve represents the asymptotic solution shown in Eq. 7, the dash curve is the numerical result based on Eq. 6, and the circles denote the result from the stochastic model. For the numerical result and the result of the stochastic model, the total number of word occurrences is fixed as 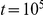. The Heaps' exponents 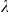 for the numerical results of Eq. 6 and the simulation results of the stochastic model are obtained by using the *least square method*.

Although Eq. 6 is different from a strict power law, numerical results indicate that the relationship between 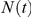 and 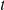 can be well fitted by the power-law functions (the fitting is usually much better than the empirical observations about the Heaps' law, see **Materials and Methods** for some typical examples). In Fig. 1, we report the numerical results with fixed total number of word occurrences 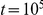. When 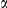 is considerably larger or smaller than 1, the numerical results agree well with the known analytical solution in Eq. 7, however, a clear deviation is observed for 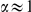 (see **Materials and Methods** about how to get the numerical results for 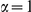).

To validate the numerical results of Eq. 6, we propose a stochastic model. Given the total number of word occurrences 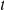, clearly, there are at most 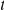 distinct words having the chance to appear. The initial occurrence number of each of these 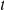 words is set as zero. At each time step, these 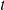 words are sorted in descending order of their occurrence number (words with the same number of occurrences are randomly ordered), and the probability a word with rank 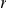 will occur in this time step is proportional to 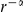. The whole process stops after 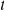 time steps. The distribution of word occurrence always obeys the Zipf's law with a stable exponent 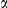, and the growth of 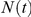 approximately follows the Heaps' law with 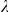 dependent on 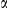 (see Figure S2 for the simulation results of the stochastic model). The simulation results about 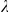 vs. 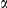 of this model are also reported in Fig. 1, which agree perfectly with the numerical ones by Eq. 6. The result of the stochastic model strongly supports the validity of Eq. 6, and thus we only discuss the numerical results of Eq. 6.

**Figure 2:**
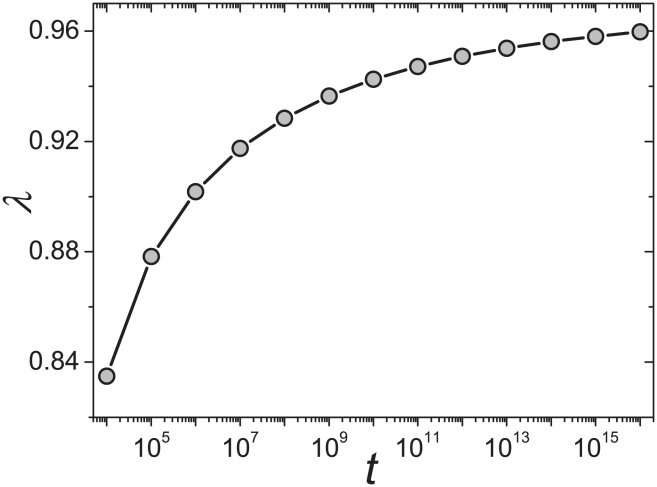
Effect of system size on the Heaps' exponent 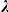. The Zipf's exponent is fixed as 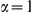.

**Figure 3:**
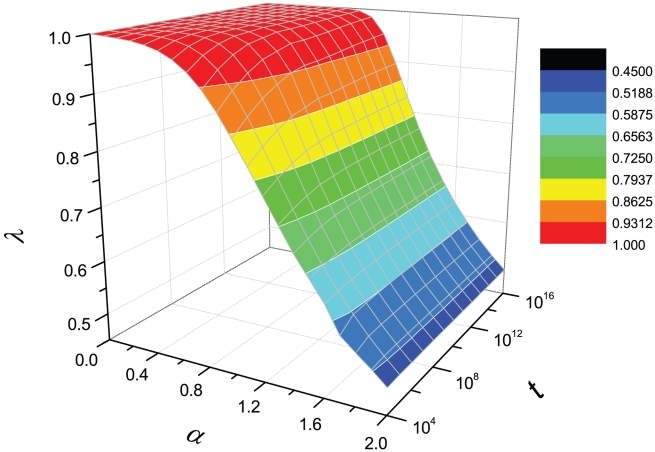
Heaps' exponent 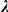 as a function of 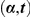.

In addition to 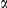, the Heaps' exponent 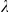 also depends on the system size, namely the total number of word occurrences, 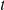. An example for 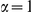 is shown in Fig. 2, and how 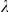 varies in the 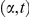 plane is shown in Fig. 3 (see Figure S3 for the comparison of fitting functions and four typical examples of numerical results). It is seen that the exponent 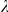 increases monotonously as the increasing of 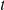. According to Eq. 6, it is obvious that in the large limit of system size, 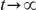, the exponent 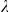 can be determined by the asymptotic solution Eq. 7. Actually, the asymptotic solution well describes the systems with 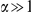 or 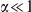 or 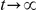. However, real systems are often with 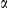 around 1 and of finite sizes. As indicated by Fig. 2 and Fig. 3, the growth of 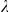 versus 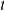 is really slow. For example, when 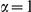, for most real systems with 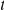 scaling from 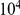 to 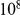, the exponent 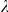 is considerably smaller than the asymptotic solution 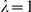. Even for very large 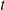 that is probably larger than any studied real systems, like 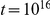, the difference between numerical result and asymptotic solution can be observed. As we will show in the next section, this paper emphasizes the difference between empirical observations and the asymptotic solution, and the simple numerical method based on Eq. 6 provides a more accurate estimation.

### Experimental Results

**Figure 4:**
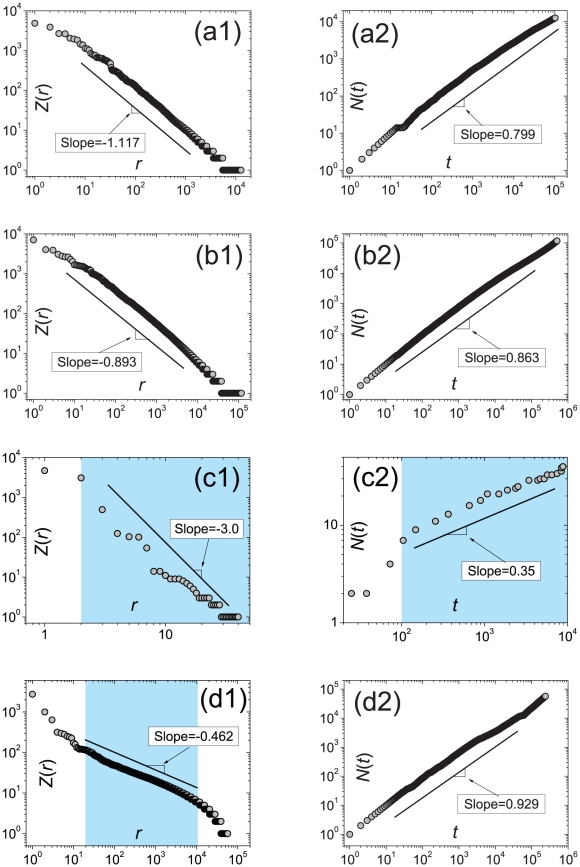
Zipf's law and Heaps' law in four example systems. (a) Words in Dante Alghieri's great book “La Divina Commedia” in Italian [44] where 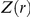 is the frequency of the word ranked 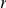 and 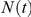 is the number of distinct words. (b) Keywords of articles published in the Proceedings of the National Academy of Sciences of the United States of America (PNAS) [30] where 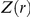 is the frequency of the keyword ranked 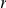 and 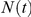 is the number of distinct keywords; (c) Confirmed cases of the novel virus influenza A (H1N1) [45] where 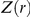 is the number of confirmed cases of the country ranked 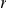 and 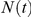 is the number of infected country in the presence of 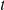 confirmed cases over the world; (d) PNAS articles having been cited at least once from 1915 to 2009 where 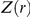 is the number of citations of the article ranked 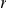 and 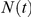 is the number of distinct articles in the presence of 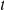 citations to PNAS. In (c), the data set is small and thus the effective number is only two digits. The fittings in (c1) and (c2) only cover the area marked by blue. In (d1), the deviation from a power law is observed in the head and tail, and thus the fitting only covers the blue area. The Zipf's (power-law) exponents and Heaps' exponents are obtained by using the *maximum likelihood estimation* [3], [43] and *least square method*, respectively. Statistics of these data sets can be found in Table 1 (the data set numbers of (a), (b), (c) and (d) are 9, 10, 34 and 35 in Table 1) with detailed description in **Materials and Methods**.

We analyze a number of real systems ranging from small-scale system containing only 40 distinct elements to large-scale system consisting of more than 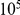 distinct elements. The results are listed in Table 1 while the detailed data description is provided in **Materials and Methods**. Four classes of real systems are considered, including the occurrences of words in different books and different languages (data sets Nos. 1–9), the occurrences of keywords in different journals (data sets Nos. 10–33), the confirmed cases of the novel virus influenza A (data set No. 34), and the citation record of PNAS articles (data set No. 35). Figure 4 reports the Zipf's law and Heaps' law of the four typical examples, each of which belongs to one class, respectively. Figure S4 in the Supporting Information displays the probability density function 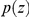, the Zipf's plot 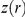 and the Heaps' plot 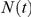 for all the 35 data sets with the same order as shown in Table 1.

**Table 1:** Empirical statistics and analysis results of real data sets.

No.	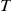	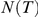	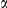	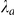	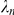	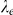
1	206779	18217	1.323	0.756	0.725	0.738
2	20516	5671	0.969	1	0.858	0.859
3	109854	13906	1.063	0.941	0.845	0.817
4	449205	20220	1.464	0.683	0.667	0.679
5	68458	9191	1.095	0.913	0.823	0.810
6	81037	13254	1.025	0.976	0.859	0.832
7	63742	16622	1.057	0.946	0.840	0.852
8	138985	15550	1.188	0.842	0.787	0.765
9	101940	12667	1.117	0.895	0.818	0.799
10	504610	116800	0.893	1	0.936	0.863
11	53214	34194	0.540	1	0.983	0.946
12	310853	69185	0.939	1	0.913	0.871
13	30852	17562	0.595	1	0.972	0.939
14	2761	2328	0.397	1	0.964	0.978
15	58300	22599	0.786	1	0.941	0.914
16	20660	8155	0.790	1	0.921	0.890
17	226090	69251	0.692	1	0.977	0.894
18	176291	62567	0.572	1	0.989	0.920
19	44735	19933	0.685	1	0.961	0.915
20	1924	1323	0.463	1	0.946	0.939
21	5093	2985	0.593	1	0.941	0.920
22	3490	2442	0.500	1	0.952	0.950
23	1403	787	0.524	1	0.926	0.931
24	7469	4142	0.654	1	0.936	0.925
25	7710	3857	0.658	1	0.935	0.930
26	3232	2658	0.416	1	0.964	0.976
27	13165	7743	0.612	1	0.959	0.936
28	3749	2353	0.568	1	0.943	0.940
29	30092	11002	0.815	1	0.924	0.891
30	21894	8666	0.776	1	0.930	0.900
31	7627	3841	0.685	1	0.933	0.930
32	4185	2242	0.675	1	0.921	0.929
33	23822	10753	0.648	1	0.959	0.917
34	8829	40	3.0	0.33	0.34	0.35
35	237982	56961	0.462	1	0.993	0.929

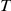
 is the total number of elements, 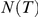 is the total number of distinct elements, 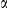 is the Zipf's exponent obtained by the *maximum likelihood estimation* [3], [43], 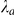 is the asymptotic solution of the Heaps' exponent as shown in Eq. 7, 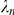 is the numerical value of the Heaps' exponent given 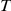 and 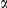 as shown in Fig. 3, and 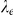 is the empirical result of the Heaps' exponent obtained by the *least square method*. The effective number of the 34th data set is only two digits since the size of this data set is very small. Except the 4th data set, in all other 34 real data sets, the numerical results based on Eq. 6 outperform the asymptotic solution shown in Eq. 7. Detailed description of these data sets can be found in **Materials and Methods**.

To sum up, the empirical results indicate that (i) evolving systems displaying the Zipf's law also obey the Heaps' law even for small-scale systems; (ii) the asymptotic solution (Eq. 7) can well capture the relationship between the Zipf's exponent and Heaps' exponent, and the present numerical result based on Eq. 6 can provide considerably better estimations (the numerical results based on Eq. 6 outperforms Eq. 7 in 34, out of 35, tested date sets).

## Discussion

Zipf's law and Heaps' law are well known in the context of complex systems. They were discovered independently and treated as two independent statistical laws for decades. Recently, the increasing evidence on the coexistence of these two laws leads to serious consideration of their relation. However, a clear picture cannot be extracted out from the literature. For example, Montemurro and Zanette [41], [42] suggested that the Zipf's law is a result from the Heaps' law while Serrano *et al.* [27] claimed that the Zipf's law can result in the Heaps' law. In addition, many previous analyses about their relation are based on some stochastic models, and the results are strongly dependent on the corresponding models – we are thus less confident of their applicability in explaining the coexistence of the two laws observed almost everywhere.

In this article, without the help of any specific stochastic model, we directly show that the Heaps' law can be considered as a derivative phenomenon given that the evolving system obeys the Zipf's law with a stable exponent. In contrast, the Zipf's law can not be derived from the Heaps' law without the help of a specific model or some external conditions. In a word, our analysis indicates that the Zipf's law is more fundamental than the Heaps' law in the systems where two laws coexist, which provides a new perspective on the origin of the Heaps' law. For example, the observed Heaps' law in natural language processing was attributed to the bursty nature and memory effect of human language [23]–[25], while Serrano, Flammini and Menczer [27] recently showed that the word occurrences in English Wikipedia also display the Heaps' law. Since the English Wikipedia is attributed by many independent editors, the memory effect is obviously not a proper interpretation. Our analysis suggests that the observed Heaps' law may be just an accompanying phenomenon of a more fundamental law – the Zipf's law. However, one can not conclude that the Heaps' law is completely dependent on the Zipf's law since there may exists some mechanisms only resulting in the Heaps' law, namely it is possible that a system displays the Heaps' law while does not obey the Zipf's law. In addition, we refine the known asymptotic solution (Eq. 7) by a more complex formula (Eq. 6), which is considerably more accurate than the asymptotic solution, as demonstrated by both the testing stochastic model and the extensive empirical analysis. In particular, our investigation about the effect of system size fills the gap in the relevant theoretical analyses.

Our analytical result (Eq. 6) indicates that the growth of vocabulary of an evolving system cannot be exactly described by the Heaps' law even though the system obeys a perfect Zipf's law with a constant exponent. In fact, not only the solution of the Heaps' exponent (Eq. 7), but also the Heaps' law itself is an asymptotic approximation obtained by considering infinite-size systems. More terribly, a Zipf's exponent larger than one does not correspond to a true distribution 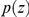 since 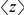 will diverge as the increasing of the system size, yet a large fraction of real systems can be well characterized by the Zipf's law with 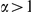 (see general examples in Refs. [3], [15] and examples of degree distributions of complex networks in Refs. [46], [47]). Putting the blemish in mathematical strictness behind, the Zipf's law and Heaps' law well capture the macroscopic statistics of many complex systems, and our analysis provides a clear picture of their relation.

Note that, our analysis depends on an ideal assumption of a “perfect” power law (Zipf's law) of frequency distribution, while a real system never displays such a perfect law. Indeed, deviations from a power law have been observed, but the assumption of a perfect power-law distribution is widely used in many theoretical analyses. For example, the degree distribution in email networks [48] has a cutoff at about 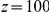 and the one in sexual contact networks [49] displays a drooping head, while in the analysis of epidemic dynamics, the underlying networks are usually supposed to be perfect scale-free networks [50]. Another example is the study on the effects of human dynamics on epidemic spreading [51], [52], where the interevent time distribution of human actions are supposed as a power-law distribution, ignoring the observed cutoffs and periodic oscillations [53], [54]. In a word, although the ideal assumption of a perfect power-law distribution could not fully reflect the reality, the corresponding analysis indeed contributes much to our understanding of many phenomena.

We also tested the power-law distribution with exponential cutoff, as 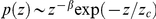, where 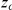 is a free parameter controlling the cutoff effect. According to the stochastic model (we first generate the rank-based distribution 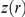 corresponding to the probability density function 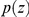, and then generate the relation 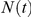 versus 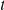 by using the stochastic model), when the cutoff effect gets enhanced (by decreasing 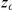), the Heaps' exponent 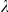 will increase (see a typical example for 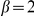 from Figure S5 in the Supporting Information). The simulation results suggest that the power-law part plays the dominant role, namely even under a very strong cutoff (e.g., 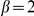 and 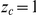, with the maximal degree is about 10), the Heaps' law still holds. But if 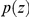 obeys an exponential form (it can even have heavier tail than the power-law distribution with strong cutoff, like 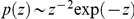), then 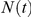 will grow almost linearly in the early stage and soon bend, deviating from the Heaps' law. The comparison of the 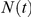 curves for power-law distribution with exponential cutoff and exponential distribution can be found in Figure S6 in the Supporting Information.

An interesting implication of our results lies in the accelerated growth of scale-free networks. Considering the degree of a node as its occurrence frequency and the total degree of all nodes as the text size, a growing network is analogous to a language system. Then, the scale-free nature corresponds to the Zipf's law of word frequency and the accelerated growth corresponds to the Heaps' law of the vocabulary growth. In an accelerated growing network, the total degree 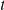 (proportional to the number of edges) scales in a power-law form as 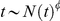, where 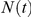 denotes the number of nodes and 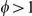 is the accelerating exponent. At the same time, the degree distribution usually follows a power law as 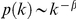 where 
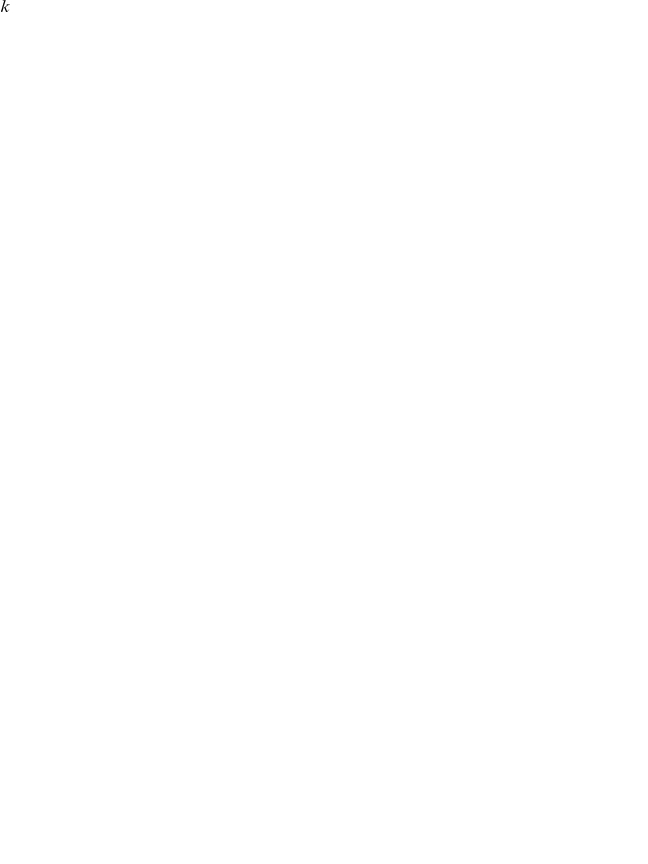
 denotes the node degree. For example, the Internet at the autonomous system (AS) level displays the scale-free nature with 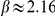 (see Table 1 in Ref. [55]) and thus 
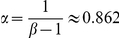
. According to a recent report [37] on empirical analysis of the Internet at the AS level, till December 2006, the total degree is 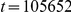. The corresponding numerical result of the Heaps' exponent is 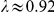 and thus the accelerating exponent can be estimated as 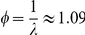. In contrast, the asymptotic solution Eq. 7 suggests a steady growing as 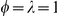. Compared with the empirical result 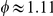 [37], Eq. 6 (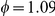) gives better result than Eq. 7 (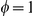). Actually, the asymptotic solution indicates that all the scale-free networks with 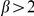 should grow in a steady (linear) manner, which is against many known empirical observations [34]–[37], while the refined result in this article is in accordance with them. Furthermore, our result provides some insights on the growth of complex networks, namely the accelerated growth can be expected if the network is scale-free with a stable exponent and this phenomenon is prominent when 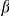 is around 2.

## Materials and Methods

### 0.1 Relation between Zipf's Law and Power Law

Given the Zipf's law 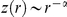, we here prove that the probability density function 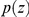 obeys a power law as 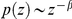 with 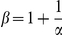. Considering the data points with ranks between 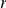 and 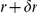 where 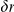 is a very small value. Clearly, the number of data points is 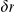, which can be expressed by the probability density function as
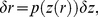
(8)where
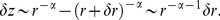
(9)Therefore, we have
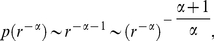
(10)namely 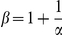. Analogously, the Zipf's law 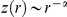 can be derived from the power-law probability density distribution 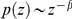, with 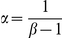.

### 0.2 Direct Comparison between Empirical and Analytical Results

**Figure 5:**
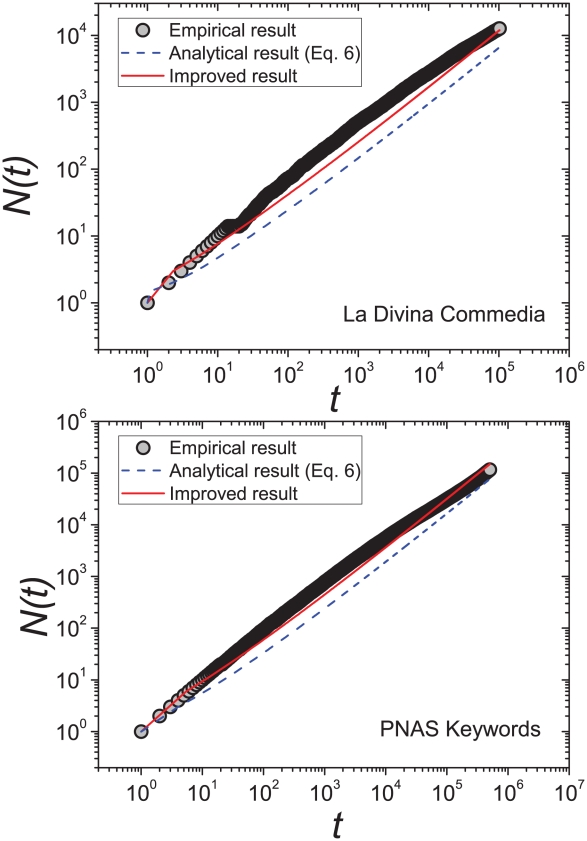
Direct comparison between the empirical data and **Eq. 6** as well as its improved version. The left and right plots are for the words in “La Divina Commedia” and the keywords in PNAS. The blue dash lines and red solid lines present the results of Eq. 6 and Eq. 11, respectively. In accordance with Figure 4 and Table 1, the values of the parameter 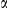 are given as 1.117 and 0.893, respectively.

Given the parameter 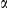, according to Eq. 6, we can numerically obtain the function 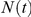. The comparison between Eq. 6 and the empirical data for words in the book “La Divina Commedia” and keywords in the PNAS articles are shown in Fig. 5. The growing tendency of distinct words can be well captured by Eq. 6. Actually, using a more accurate normalization condition 
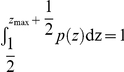
, as an improved version of Eq. 4, the estimation of 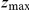 is determined by
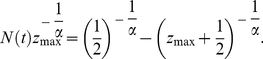
(11)


Given the parameter 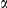, for an arbitrary 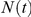, one can estimate the corresponding 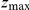 according to Eq. 11 and then determine the value of 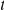 by Eq. 5. The numerical results of this improved version are also presented in Fig. 5, which fits better than Eq. 6 to the empirical data. Notice that, both the two analytical results give almost the same slope in the log-log plot of 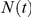 function, namely the Heaps' exponents obtained by these two versions are almost the same.

### 0.3 Examples of Numerical Results

**Figure 6:**
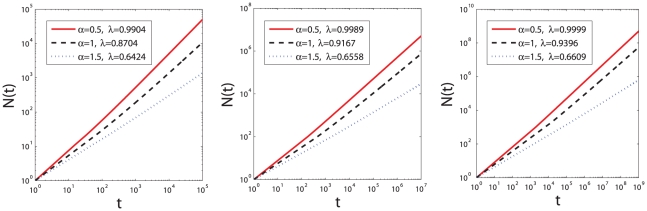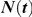 vs. 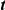 according to the numerical results of Eq. 6. The red, black and blue line correspond to the cases of 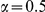, 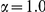 and 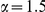. The system sizes (i.e., the total number of word occurrences), from left to right, are 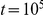, 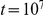 and 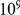. Fitting exponent 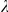 is obtained by the *least square method*. The fitting lines and numerical results almost completely overlap.

Mathematically speaking, as indicated by Eq. 6, 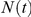 does not scale in a power law with 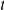. However, the numerical results suggest that the dependence of 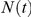 on 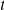 can be well approximated as power-law functions. As shown in Fig. 6, for a wide range of 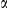, 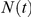 can be well fitted by 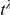, and the value of fitting exponent 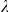 depends on both 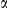 and 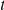.

### 0.4 The case of 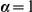


The numerical solution of Eq. 6 for 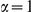 can be obtained by considering the limitation 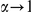, where 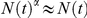 and 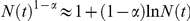. Accordingly, Eq. 6 can be rewritten as
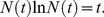
(12)When 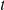 approaches to infinity, 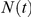 scales almost linearly with 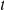 since 
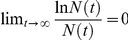
. Actually, the solution can be expressed as 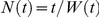 where 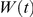 is the well-known *Lambert W function* [56] that satisfies
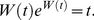
(13)For any finite system, the numerical result can be produced by Eq. 12.

### 0.5 Data description

The data sets analyzed in this article can be divided into four classes. According to the data sets shown in Table 1, these four classes are as follows.

(i) Occurrences of words in different books and different languages (data sets Nos. 1–9). The data set No. 1 is the English book (*Moby Dick*) written by Herman Melville; the data sets No. 2 (*De Bello Gallico*), No. 3 (*Philosophiæ Naturalis Principia Mathematica*) and No. 7 (*Aeneis*) are Latin books written by Gaius Julius Caesar, Isaac Newton and Virgil respectively; the data sets No. 4 (*Don Quijote*), No. 5 (*La Celestina*) and No. 8 (*Cien a* 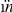 *os de soledad*) are Spanish novels written by Miguel de Cervantes, Fernando de Rojas and Gabriel García Márquez, respectively; the data set No. 6 (*Faust*) is a German opera written by Johann Wolfgang von Goethe; the data set No. 9 (*La Divina Commedia di Dante*) is the Italian epic poem written by Dante Alighieri. All the above data are collected by Carpena *et al.* [44] and available at http://bioinfo2.ugr.es/TextKeywords/index.html.

(ii) Occurrences of keywords in different journals (data sets Nos. 10–33). These 24 journals, from No. 10 to No. 33 are PNAS, Chin. Sci. Bull., J. Am. Chem. Soc., Acta Chim. Sinica, Crit. Rev. Biochem. Mol. Biol., J. Biochem., J. Nutr. Biochem., Phys. Rev. Lett., Appl. Phys. Lett., Physica A, ACM Comput. Surv., ACM Trans. Graph., Comput. Netw., ACM Trans. Comput. Syst., Econmetrica, J. Econ. Theo., SIAM Rev., SIAM J. Appl. Math., Invent. Math., Ann. Neurol., J. Evol. Biol., Theo. Popul. Biol., MIS Quart., and IEEE Trans. Automat. Contr.. These data are collected from the ISI Web of Knowledge (http://isiknowledge.com/). For every scientific journal, we consider the keywords sequence in each article according to its publishing time. Since most of the published articles do not have keywords before 1990 in ISI database, we limit our collections from 1991 to 2007 (except for ACM Comput. Surv. which is available only from 1994 to 1999).

(iii) Confirmed cases of the novel virus influenza A in 2009 (data set No. 34). The data of the cumulative number of laboratory confirmed cases of H1N1 of each country are available from the website of Epidemic and Pandemic Alert of World Health Organization (WHO) (http://www.who.int/). The analyzed data set reported influenza A starting from April 26th to May 18th, updated each one or two days. After May 18th, the distribution of confirmed cases in each country shifted from a power law to a power-law form with exponential cutoff [45].

(iv) Citation record of PNAS articles (data set No. 35). This data set consists of all the citations to PNAS articles from papers published between 1915 and 2009 according to the ISI database, ordered by time.

## Supporting Information

Figure S1 Relative errors of the approximation in Eq. 5.
(0.08 MB PDF)

Figure S2 Zipf's law and Heaps' law resulted from the stochastic model.
(0.48 MB PDF)

Figure S3 Fitting Heaps' law with different system sizes.
(1.04 MB PDF)

Figure S4 Probability density functions, rank-based distributions (Zipf's plots) and Heaps' plots for all the 35 data sets shown in Table 1.
(1.29 MB PDF)

Figure S5 Effects of exponential cutoffs on the Heaps' law.
(1.68 MB PDF)

Figure S6 Comparison of the growing tendencies of $N(t)$ between the cases of power-law distribution with an exponential cutoff and the purely exponential distribution.
(0.09 MB PDF)

## References

[1] Zipf GK (1949). Human Behaviour and the Principle of Least Effort: An introduction to human ecology (Addison-Wesly, Cambridge).

[2] Heaps HS (1978). Information Retrieval: Computational and Theoretical Aspects (Academic Press, Orlando).

[3] Clauset A, Shalizi CR, Newman MEJ (2009). Power-law distributions in empirical data.. SIAM Rev.

[4] Axtell RL (2001). Zipf Distribution of U.S. Firm Sizes.. Science.

[5] Drăgulescu A, Yakovenko VM (2001). Exponential and power-law probability distributions of wealth and income in the United Kingdom and the United States.. Physica A.

[6] Redner S (1998). How popular is your paper? An empirical study of the citation distribution.. Eur Phys J B.

[7] Furusawa C, Kaneko K (2003). Zipf's Law in Gene Expression.. Phys Rev Lett.

[8] Bai W-J, Zhou T, Fu Z-Q, Chen Y-H, Wu X, Wang BH (2006). Electric power grids and blackouts in perspective of complex networks.. Proc. 4th International Conference on Communications, Circuits and Systems (IEEE Press, New York).

[9] Baek SK, Kiet HAT, Kim BJ (2007). Family name distributions: Master equation approach.. Phys Rev E.

[10] Cordoba JC (2008). On the distribution of city sizes.. J Urban Econ.

[11] Chen Q, Wang C, Wang Y (2009). Deformed Zipf's law in personal donation.. EPL.

[12] Blasius B, Tönjes R (2009). Zipf's Law in the Popularity Distribution of Chess Openings.. Phys Rev Lett.

[13] Abhari A, Soraya M (2010). Workload generation for YouTube.. Multimedia Tools Appl.

[14] Mitzenmacher M (2004). A brief history of generative models for power law and lognormal distributions.. Internet Mathematics.

[15] Newman MEJ (2005). Power laws, Pareto distributions and Zipf's law.. Contemporary Physics.

[16] Simon HA (1955). On a class of skew distribution functions.. Biometrika.

[17] Barabasi A-L, Albert R (1999). Emergence of scaling in random networks.. Science.

[18] Bak P (1996). How Nature Works: The Science of Self-Organized Criticality (Copernicus, New York).

[19] Kanter I, Kessler DA (1995). Markov Processes: Linguistics and Zipf's Law.. Phys Rev Lett.

[20] Marsili M, Zhang Y-C (1996). Interacting Individuals Leading to Zipf's Law.. Phys Rev Lett.

[21] Carlson JM, Doyle J (1999). Highly optimized tolerance: A mechanism for power laws in designed systems.. Phys Rev E.

[22] Cancho RFi, Solé RV (2003). Least effort and the origins of scaling in human language.. Proc Natl Acad Sci USA.

[23] Ebeling W, Pöschel T (1994). Entropy and long range correlations in literary English.. Europhys Lett.

[24] Kleinberg J (2003). Bursty and Hierarchical Structure in Streams.. Data Min Knowl Disc.

[25] Altmann EG, Pierrehumbert JB, Motter AE (2009). Beyond Word Frequency: Bursts, Lulls, and Scaling in the Temporal Distributions of Words.. PLoS ONE.

[26] Gelbukh A, Sidorov G (2001). Zipf and Heaps Laws' Coefficients Depend on Language.. Lect Notes Comput Sci.

[27] Serrano MÁ, Flammini A, Menczer F (2009). Modeling Statistical Properties of Written Text.. PLoS ONE.

[28] Cattuto C, Loreto V, Pietronero L (2007). Semiotic dynamics and collaborative tagging,. Proc Natl Acad Sci USA.

[29] Cattuto C, Barrat A, Baldassarri A, Schehr G, Loreto V (2009). Collective dynamics of social annotation.. Proc Natl Acad Sci USA.

[30] Zhang Z-K, Lü L, Liu J-G, Zhou T (2008). Empirical analysis on a keyword-based semantic system.. Eur Phys J B.

[31] Lansey JC, Bukiet B (2009). Internet Search Result Probabilities: Heaps' Law and Word Associativity.. J Quant Linguistics.

[32] Zhang H-Y (2009). Discovering power laws in computer programs.. Inf Process Manage.

[33] Benz RW, Swamidass SJ, Baldi P (2008). Discovery of Power-Laws in Chemical Space.. J Chem Inf Model.

[34] Dorogovtsev SN, Mendes JFF (2001). Effect of the accelerating growth of communications networks on their structure.. Phys Rev E.

[35] Smith DMD, Onnela J-P, Johnson NF (2007). Accelerating networks.. New J Phys.

[36] Broder A, Kumar R, Moghoul F, Raghavan P, Rajagopalan S, Stata R, Tomkins A, Wiener J (2000). Graph structure in the Web.. Comput Netw.

[37] Zhang G-Q, Zhang G-Q, Yang Q-F, Cheng S-Q, Zhou T (2008). Evolution of the Internet and its cores.. New J Phys.

[38] Baeza-Yates RA, Navarro G (2000). Block addressing indices for approximate text retrieval.. J Am Soc Inf Sci.

[39] van Leijenhorst DC, Weide Th P van der (2005). A formal derivation of Heaps' Law.. Inf Sci.

[40] Mandelbrot B (1960). The pareto-levy law and the distribution of income.. Int Econo Rev.

[41] Montemurro MA, Zanette DH (2002). New prespectives on Zipf's law in linguistics: from single texts to large corpora.. Glottometrics.

[42] Zanette DH, Montemurro MA (2005). Dynamics of Text Generation with Realistic Zipf's Distribution.. J Quant Linguistics.

[43] Goldstein ML, Morris SA, Yen GG (2004). Problems with fitting to the power-law distribution.. Eur Phys J B.

[44] Carpena P, Bernaola-Galván P, Hackenberg M, Coronado AV, Oliver JL (2009). Level statistics of words: Finding keywords in literary texts and symbolic sequences.. Phys Rev E.

[45] Han XP, Wang BH, Zhou CS, Zhou T, Zhu JF (2009). Scaling in the Global Spreading Patterns of Pandemic Influenza A and the Role of Control: Empirical Statistics and Modeling, e-print arXiv.

[46] Albert R, Barabási A-L (2002). Statistical mechanics of complex networks.. Rev Mod Phys.

[47] Newman MEJ (2003). The structure and function of complex networks.. SIAM Rev.

[48] Ebel H, Mielsch L-I, Bornholdt S (2002). Scale-free topology of e-mail networks.. Phys Rev E.

[49] Liljeros F, Edling CR, Amaral LAN, Stanley HE, Åberg Y (2001). The web of human sexual contacts.. Nature.

[50] Pastor-Satorras R, Vespignani A (2001). Epidemic Spreading in Scale-Free Networks.. Phys Rev Lett.

[51] Vázquez A, Rácz B, Lukács A, Barabási A-L (2007). Impact of Non-Poissonian Activity Patterns on Spreading Processes.. Phys Rev Lett.

[52] Iribarren JL, Moro E (2009). Impact of Human Activity Patterns on the Dynamics of Information Diffusion.. Phys Rev Lett.

[53] Zhou T, Kiet HAT, Kim BJ, Wang BH, Holme P (2008). Role of activity in human dynamics.. EPL.

[54] Radicchi F (2009). Human activity in the web.. Phys Rev E.

[55] Caldarelli G (2007). Scale-Free Networks: Complex webs in nature and technology (Oxford University Press, Oxford).

[56] Corless RM, Gonnet GH, Hare DEG, Jeffrey DJ, Knuth DE (1996). On the Lambert W function.. Adv Comput Math.

