# Supplementary figures and images for: Zipf's Law Leads to Heaps' Law: Analyzing Their Relation in Finite-Size Systems
Source: PLoS One. 2010 Dec 2;5(12):e14139. doi: 10.1371/journal.pone.0014139 (PMC2996287; doi:10.1371/journal.pone.0014139)

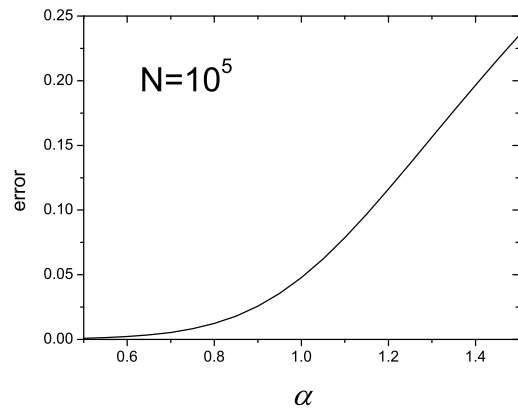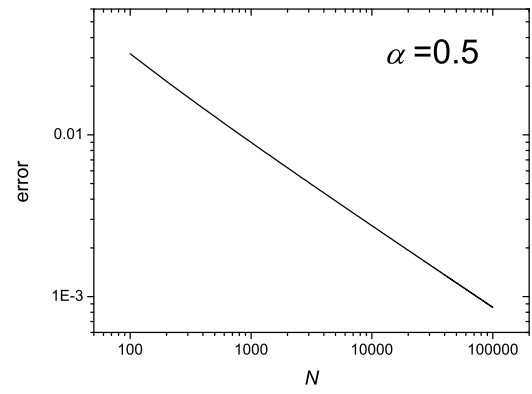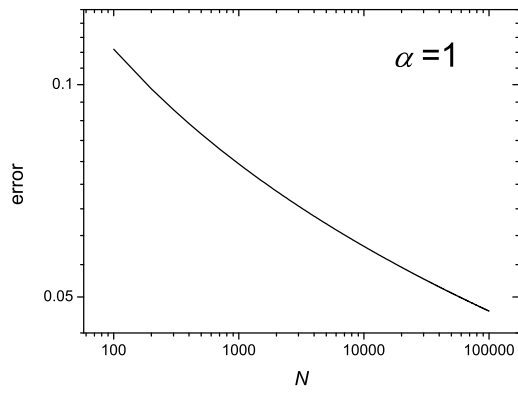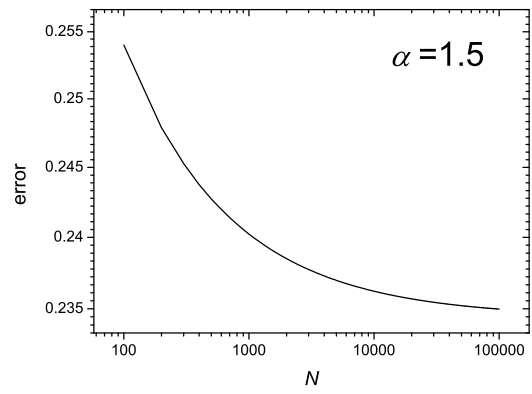

**Figure S1: Relative errors of the approximation in Eq. 5.**

Supplement: Figure S1 — Relative errors of the approximation in Eq. 5. (0.08 MB PDF) [file pone.0014139.s001.pdf]
