# Supplementary material for: Zipf's Law Leads to Heaps' Law: Analyzing Their Relation in Finite-Size Systems
Source: PLoS One. 2010 Dec 2;5(12):e14139. doi: 10.1371/journal.pone.0014139 (PMC2996287; doi:10.1371/journal.pone.0014139)
Supplement: Figure S2 — Zipf's law and Heaps' law resulted from the stochastic model. (0.48 MB PDF) [file pone.0014139.s002.pdf]

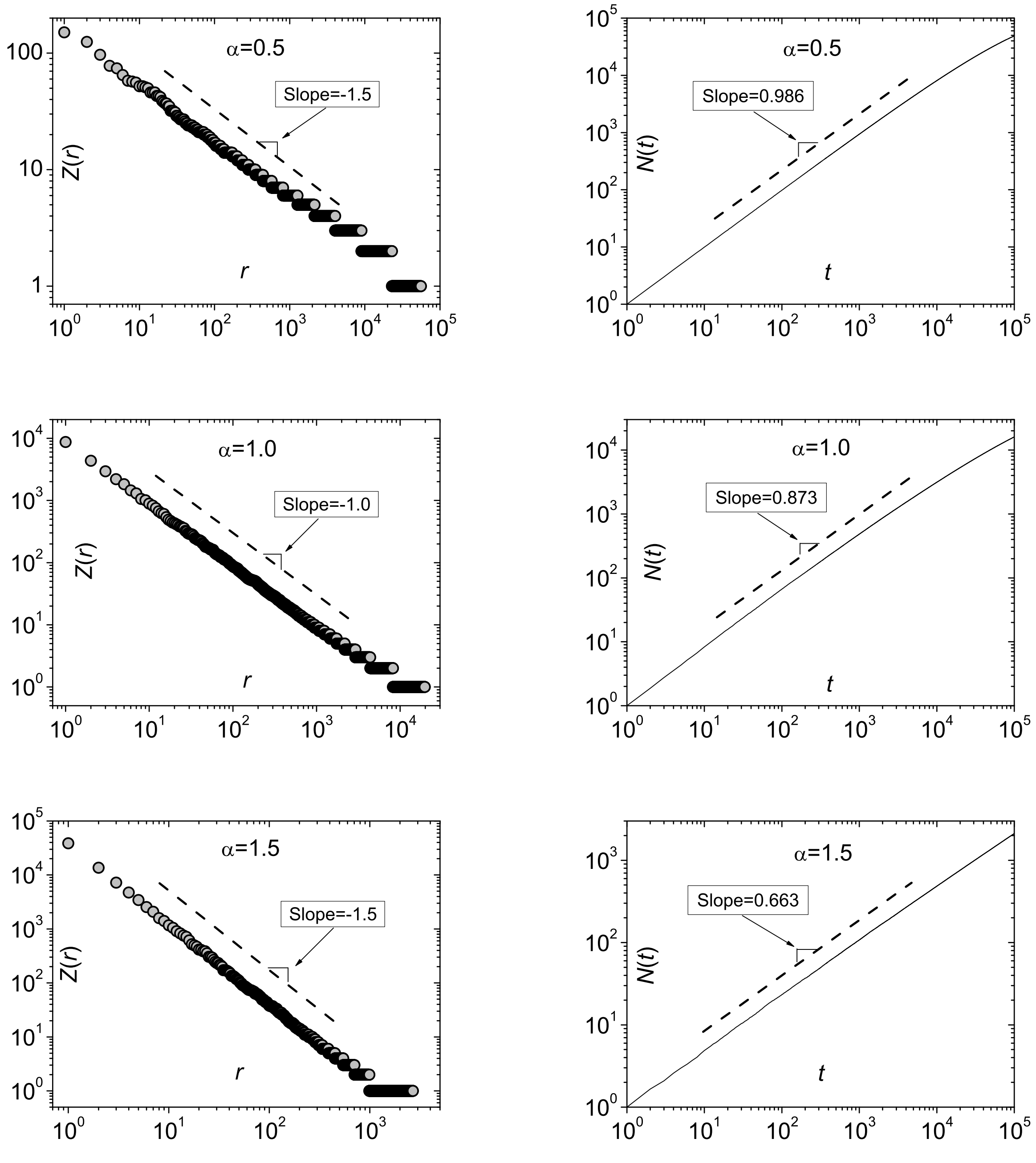

Figure S2: Zipf's law and Heaps' law resulted from the stochastic model. The six plots display three typical examples for $\alpha = 0.5$, $\alpha = 1.0$ and $\alpha = 1.5$. The slopes of Zipf's plot and Heaps' plot are respectively obtained by the maximum likelihood method and the least square method. The simulation results agree well with the theoretical expectations.
