# Supplementary material for: Zipf's Law Leads to Heaps' Law: Analyzing Their Relation in Finite-Size Systems
Source: PLoS One. 2010 Dec 2;5(12):e14139. doi: 10.1371/journal.pone.0014139 (PMC2996287; doi:10.1371/journal.pone.0014139)
Supplement: Figure S3 — Fitting Heaps' law with different system sizes. (1.04 MB PDF) [file pone.0014139.s003.pdf]

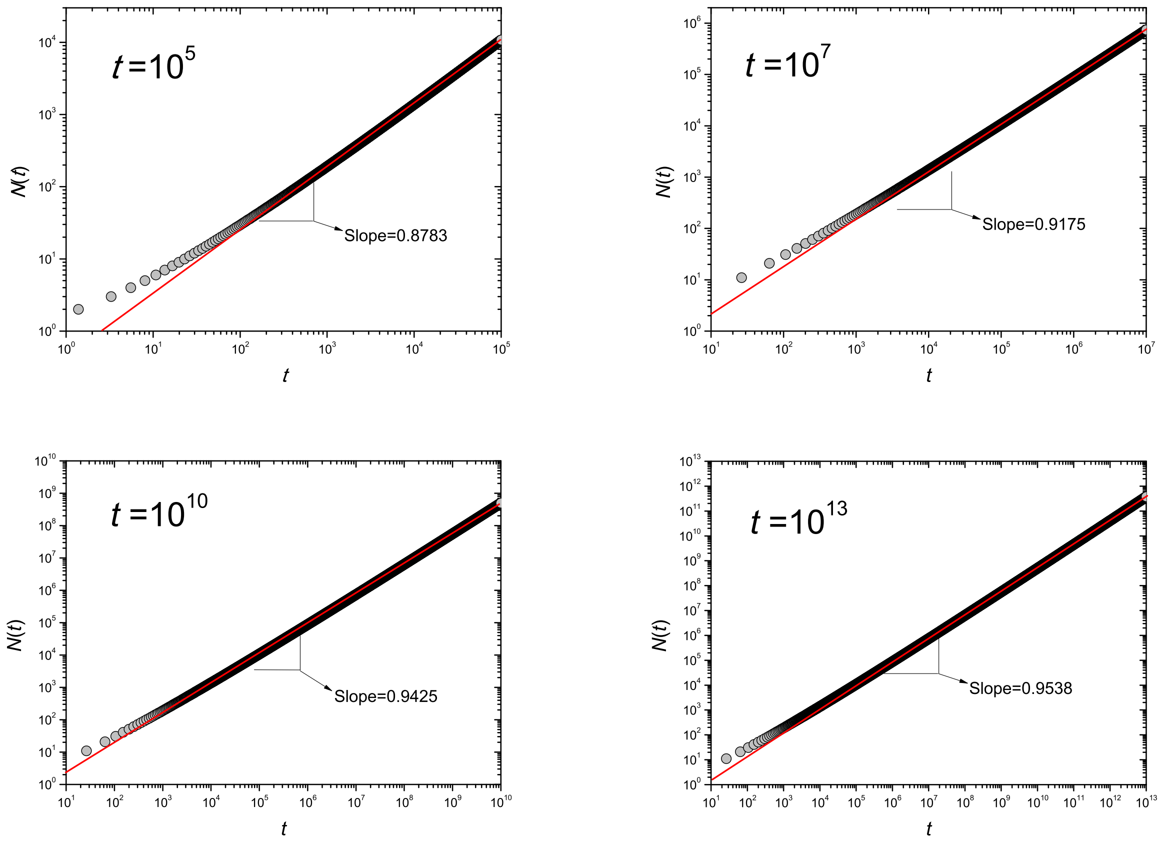

Figure S3: Fitting Heaps' law with different system sizes. The four plots show typical examples with different system sizes t, with $\alpha = 1$ fixed. The fitting functions are obtained by using the linear regression with the least square method, which well capture the growing tendency of $N(t)$.
