# Supplementary material for: Zipf's Law Leads to Heaps' Law: Analyzing Their Relation in Finite-Size Systems
Source: PLoS One. 2010 Dec 2;5(12):e14139. doi: 10.1371/journal.pone.0014139 (PMC2996287; doi:10.1371/journal.pone.0014139)
Supplement: Figure S4 — Probability density functions, rank-based distributions (Zipf's plots) and Heaps' plots for all the 35 data sets shown in Table 1. (1.29 MB PDF) [file pone.0014139.s004.pdf]

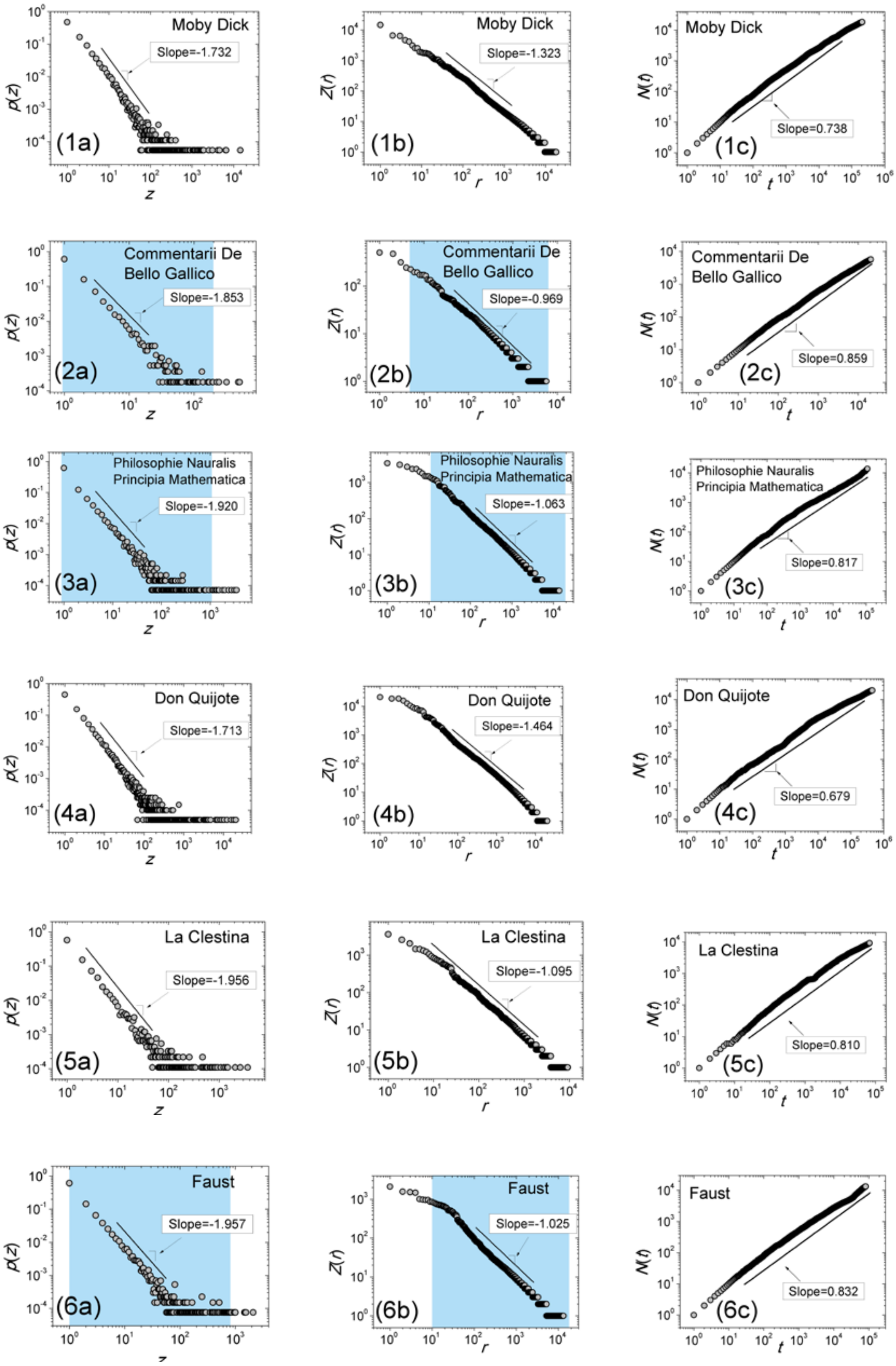
Moby Dick
Slope=-1.732
(1a)
p(z)
z
Moby Dick
Slope=-1.323
(1b)
Z(r)
r
Moby Dick
Slope=0.738
(1c)
N(t)
t
Commentarii De Bello Gallico
Slope=-1.853
(2a)
Commentarii De Bello Gallico
Slope=-0.969
(2b)
Commentarii De Bello Gallico
Slope=0.859
(2c)
Philosophie Nauralis Principia Mathematica
Slope=-1.920
(3a)
Philosophie Nauralis Principia Mathematica
Slope=-1.063
(3b)
Philosophie Nauralis Principia Mathematica
Slope=0.817
(3c)
Don Quijote
Slope=-1.713
(4a)
Don Quijote
Slope=-1.464
(4b)
Don Quijote
Slope=0.679
(4c)
La Clestina
Slope=-1.956
(5a)
La Clestina
Slope=-1.095
(5b)
La Clestina
Slope=0.810
(5c)
Faust
Slope=-1.957
(6a)
Faust
Slope=-1.025
(6b)
Faust
Slope=0.832
(6c)

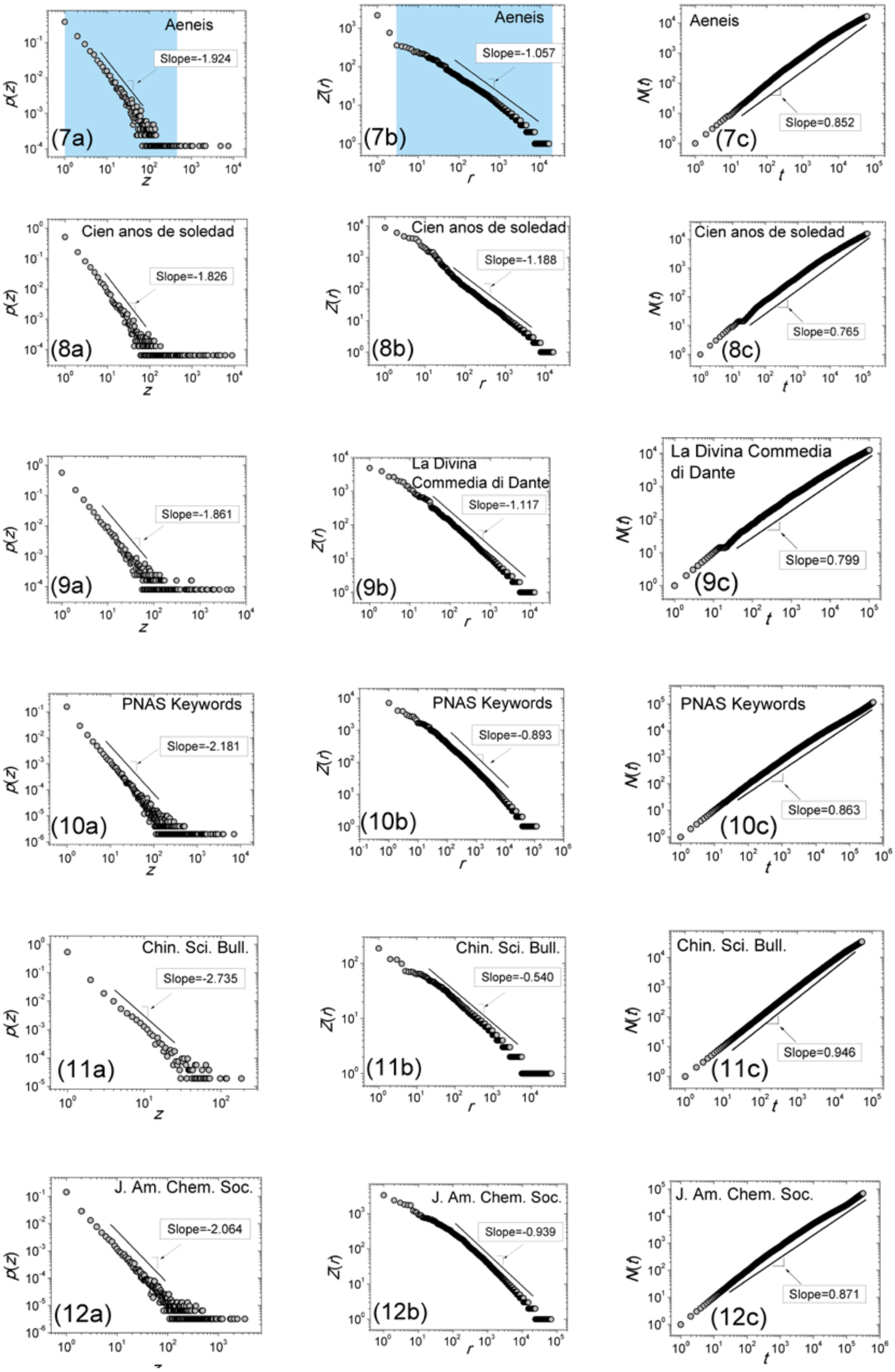

Aeneis
Slope=-1.924
(7a)
Aeneis
Slope=-1.057
(7b)
Aeneis
Slope=0.852
(7c)
Cien anos de soledad
Slope=-1.826
(8a)
Cien anos de soledad
Slope=-1.188
(8b)
Cien anos de soledad
Slope=0.765
(8c)
Slope=-1.861
(9a)
La Divina Commedia di Dante
Slope=-1.117
(9b)
La Divina Commedia di Dante
Slope=0.799
(9c)
PNAS Keywords
Slope=-2.181
(10a)
PNAS Keywords
Slope=-0.893
(10b)
PNAS Keywords
Slope=0.863
(10c)
Chin. Sci. Bull.
Slope=-2.735
(11a)
Chin. Sci. Bull.
Slope=-0.540
(11b)
Chin. Sci. Bull.
Slope=0.946
(11c)
J. Am. Chem. Soc.
Slope=-2.064
(12a)
J. Am. Chem. Soc.
Slope=-0.939
(12b)
J. Am. Chem. Soc.
Slope=0.871
(12c)
p(z)
z
Z(r)
r
N(t)
t

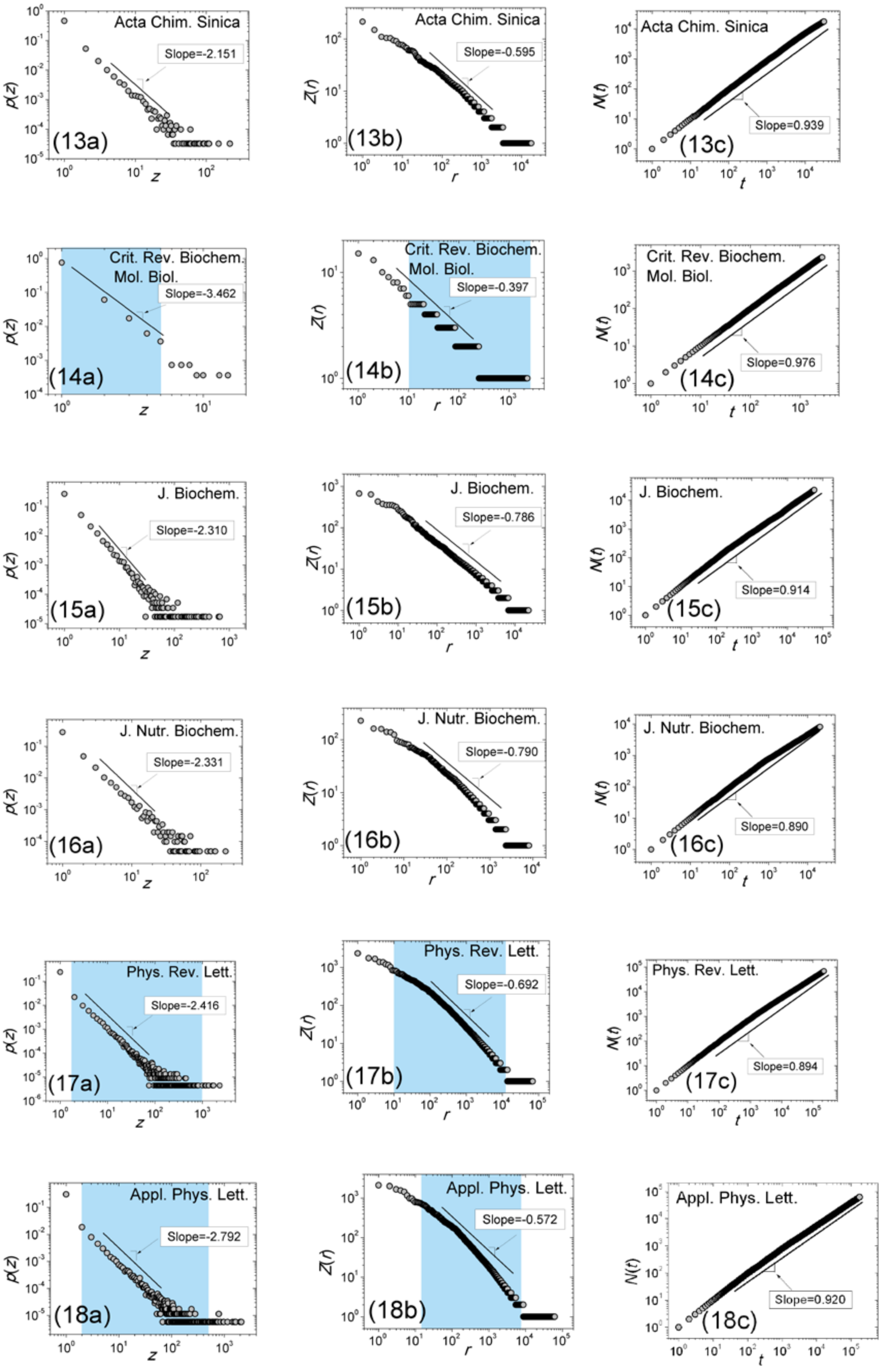

Acta Chim. Sinica
Slope=-2.151
(13a)
Acta Chim. Sinica
Slope=-0.595
(13b)
Acta Chim. Sinica
Slope=0.939
(13c)
Crit. Rev. Biochem. Mol. Biol.
Slope=-3.462
(14a)
Crit. Rev. Biochem. Mol. Biol.
Slope=-0.397
(14b)
Crit. Rev. Biochem. Mol. Biol.
Slope=0.976
(14c)
J. Biochem.
Slope=-2.310
(15a)
J. Biochem.
Slope=-0.786
(15b)
J. Biochem.
Slope=0.914
(15c)
J. Nutr. Biochem.
Slope=-2.331
(16a)
J. Nutr. Biochem.
Slope=-0.790
(16b)
J. Nutr. Biochem.
Slope=0.890
(16c)
Phys. Rev. Lett.
Slope=-2.416
(17a)
Phys. Rev. Lett.
Slope=-0.692
(17b)
Phys. Rev. Lett.
Slope=0.894
(17c)
Appl. Phys. Lett.
Slope=-2.792
(18a)
Appl. Phys. Lett.
Slope=-0.572
(18b)
Appl. Phys. Lett.
Slope=0.920
(18c)

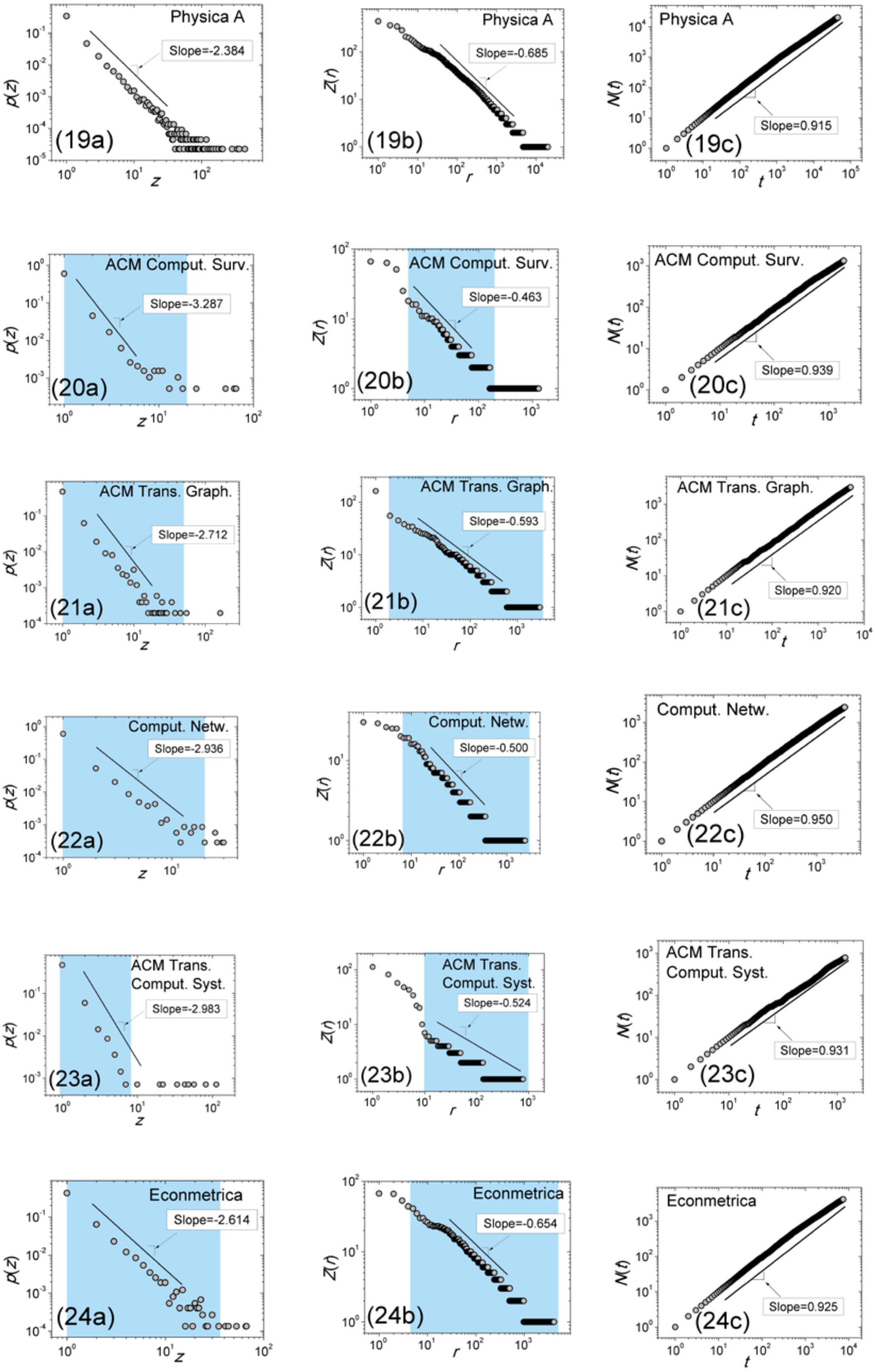

Physica A
Slope=-2.384
(19a)
p(z)
z
Physica A
Slope=-0.685
(19b)
Z(r)
r
Physica A
Slope=0.915
(19c)
N(t)
t
ACM Comput. Surv.
Slope=-3.287
(20a)
ACM Comput. Surv.
Slope=-0.463
(20b)
ACM Comput. Surv.
Slope=0.939
(20c)
ACM Trans. Graph.
Slope=-2.712
(21a)
ACM Trans. Graph.
Slope=-0.593
(21b)
ACM Trans. Graph.
Slope=0.920
(21c)
Comput. Netw.
Slope=-2.936
(22a)
Comput. Netw.
Slope=-0.500
(22b)
Comput. Netw.
Slope=0.950
(22c)
ACM Trans. Comput. Syst.
Slope=-2.983
(23a)
ACM Trans. Comput. Syst.
Slope=-0.524
(23b)
ACM Trans. Comput. Syst.
Slope=0.931
(23c)
Econmetrica
Slope=-2.614
(24a)
Econmetrica
Slope=-0.654
(24b)
Econmetrica
Slope=0.925
(24c)

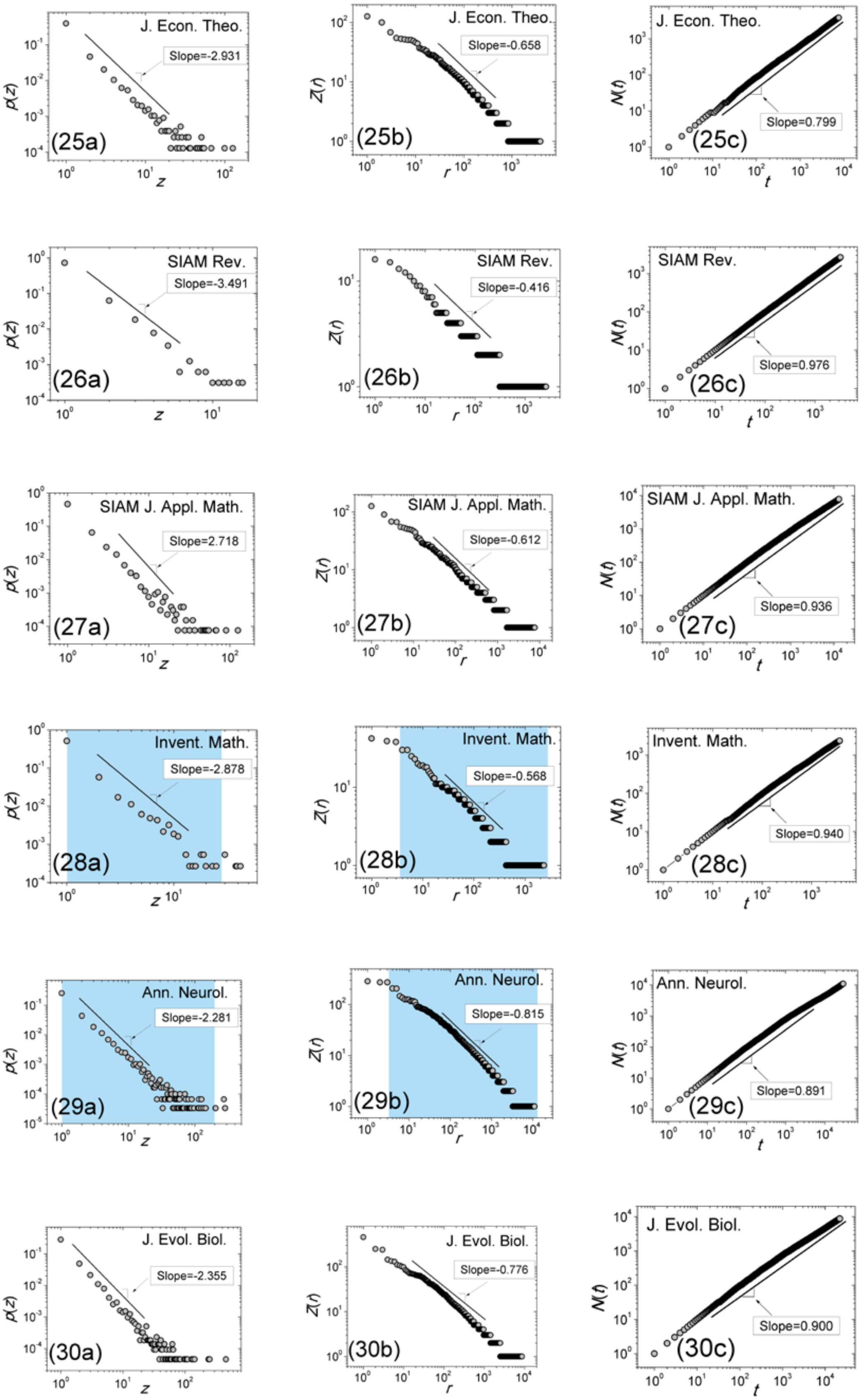

J. Econ. Theo.
Slope=-2.931
(25a)
J. Econ. Theo.
Slope=-0.658
(25b)
J. Econ. Theo.
Slope=0.799
(25c)
SIAM Rev.
Slope=-3.491
(26a)
SIAM Rev.
Slope=-0.416
(26b)
SIAM Rev.
Slope=0.976
(26c)
SIAM J. Appl. Math.
Slope=2.718
(27a)
SIAM J. Appl. Math.
Slope=-0.612
(27b)
SIAM J. Appl. Math.
Slope=0.936
(27c)
Invent. Math.
Slope=-2.878
(28a)
Invent. Math.
Slope=-0.568
(28b)
Invent. Math.
Slope=0.940
(28c)
Ann. Neurol.
Slope=-2.281
(29a)
Ann. Neurol.
Slope=-0.815
(29b)
Ann. Neurol.
Slope=0.891
(29c)
J. Evol. Biol.
Slope=-2.355
(30a)
J. Evol. Biol.
Slope=-0.776
(30b)
J. Evol. Biol.
Slope=0.900
(30c)
p(z)
z
Z(r)
r
N(t)
t

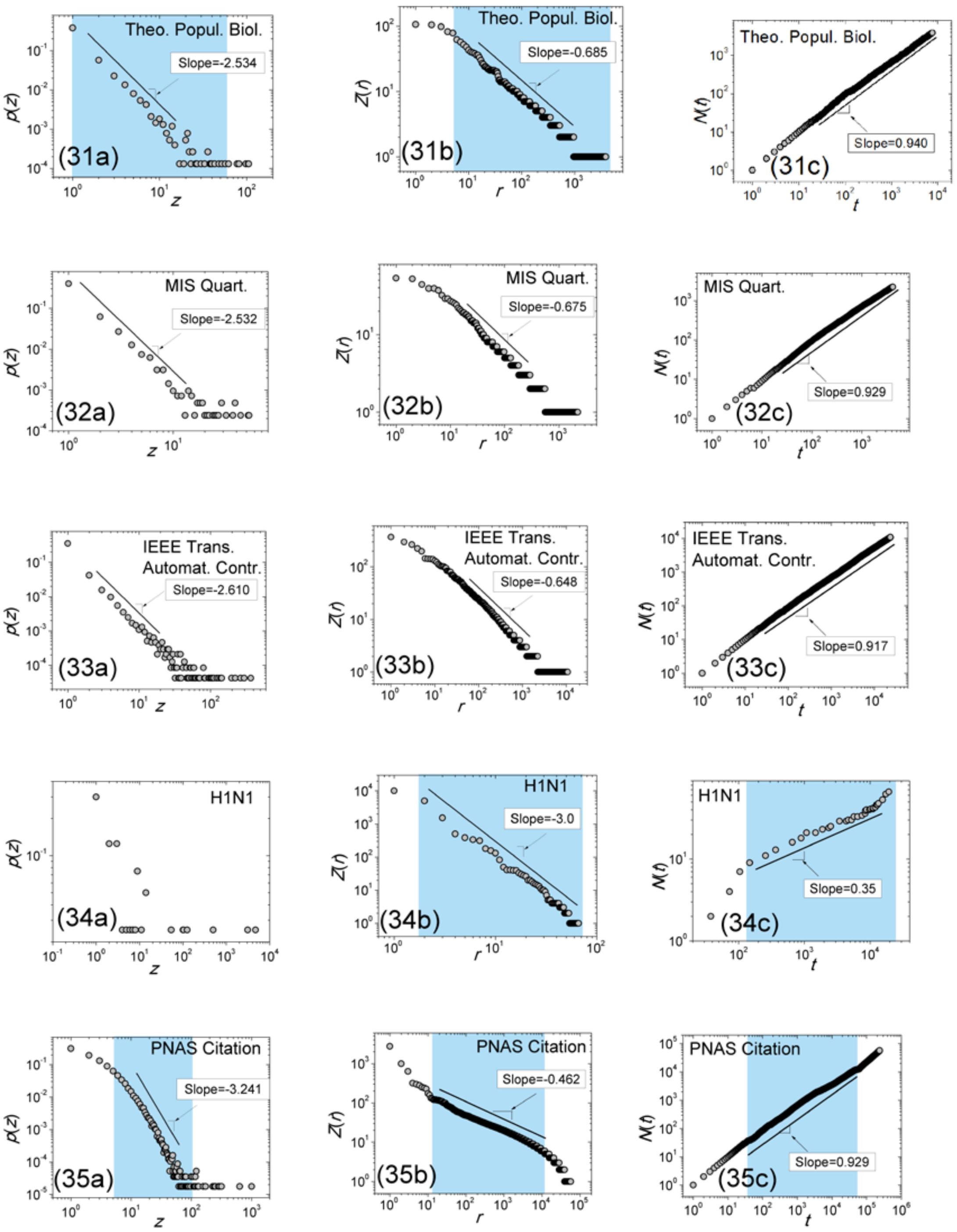

Figure S4: **Probability density functions, rank-based distributions (Zipf's plots) and Heaps' plots for all the 35 data sets shown in Table 1**. The indices in the plots are exactly the same as the ones in Table 1. Some data sets are small, and thus the fittings can only cover the blue areas. Especially the H1N1 data set (No. 34) is very small and thus only the Zipf's plot can provide some statistics, while the probability density function cannot be well fitted.
