# Supplementary material for: Zipf's Law Leads to Heaps' Law: Analyzing Their Relation in Finite-Size Systems
Source: PLoS One. 2010 Dec 2;5(12):e14139. doi: 10.1371/journal.pone.0014139 (PMC2996287; doi:10.1371/journal.pone.0014139)
Supplement: Figure S5 — Effects of exponential cutoffs on the Heaps' law. (1.68 MB PDF) [file pone.0014139.s005.pdf]

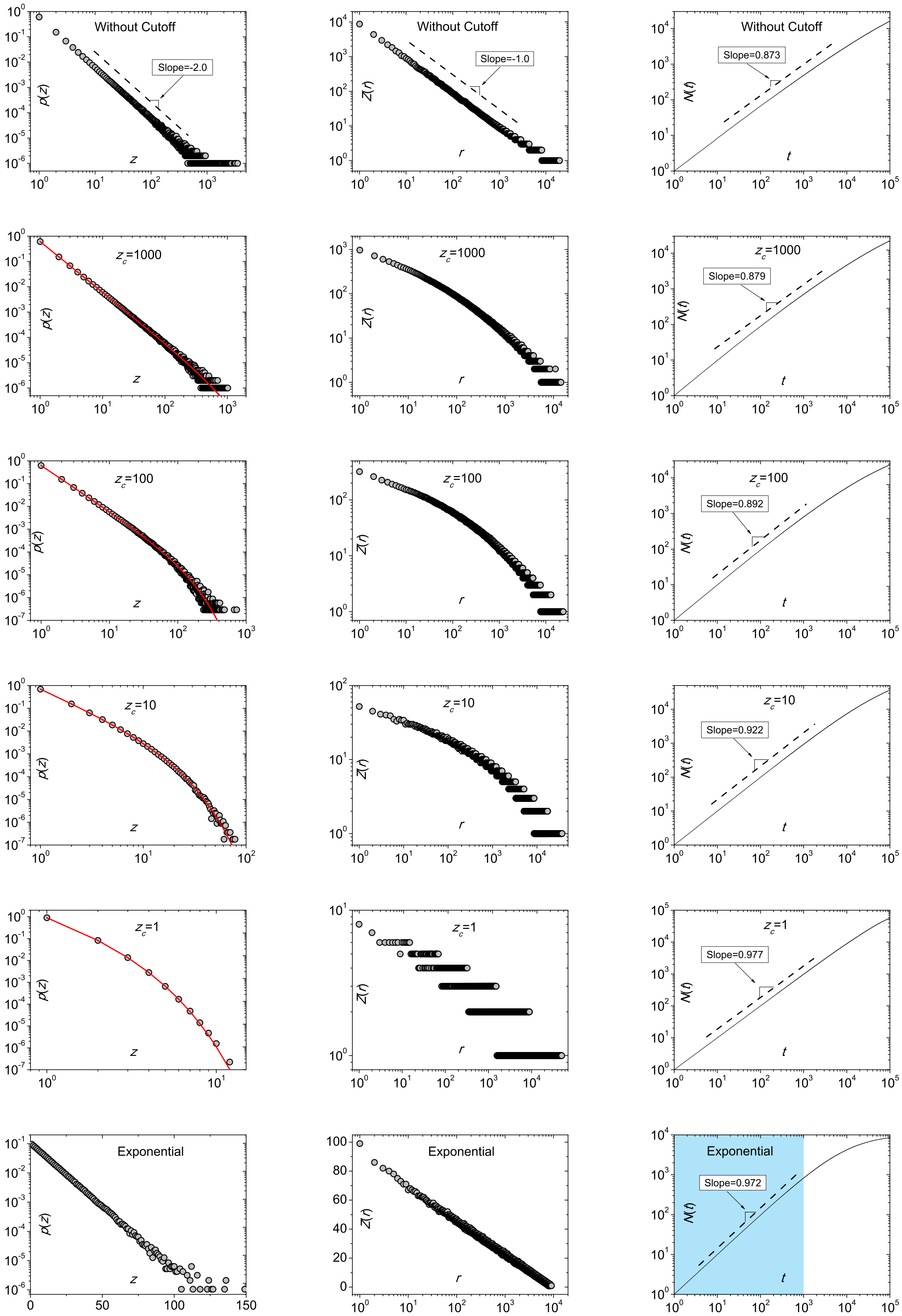

Figure S5: Effects of exponential cutoffs on the Heaps' law. All the plots are generated by the stochastic model with $\alpha = 1$ (i.e., $\beta = 2$). Instead of a prefect power-law distribution, here the probability density function $p(z)$ obeys the form $p(z) \sim z^{-2}\exp(-z/z_c)$. The three plots in the first line corresponds to the case without cutoff (i.e., $z_c \to \infty$). The following four lines correspond to $z_c = 1000$, $z_c = 100$, $z_c = 10$ and $z_c = 1$, respectively. Clearly, as the decreasing of z_c (i.e., the enhancing of cutoff effect), the Heaps' law still holds with an increasing exponent. The last line shows the result for purely exponential distribution $p(z) \sim \exp(-z/10)$. Although this distribution has heavier tail than the one with $z_c = 1$, it does not obey the Heaps' law. Actually, it grows almost linearly in the blue area (the fitting slope of the blue area is 0.972) but soon bends.
