# Supplementary material for: Zipf's Law Leads to Heaps' Law: Analyzing Their Relation in Finite-Size Systems
Source: PLoS One. 2010 Dec 2;5(12):e14139. doi: 10.1371/journal.pone.0014139 (PMC2996287; doi:10.1371/journal.pone.0014139)
Supplement: Figure S6 — Comparison of the growing tendencies of $N(t)$ between the cases of power-law distribution with an exponential cutoff and the purely exponential distribution. (0.09 MB PDF) [file pone.0014139.s006.pdf]

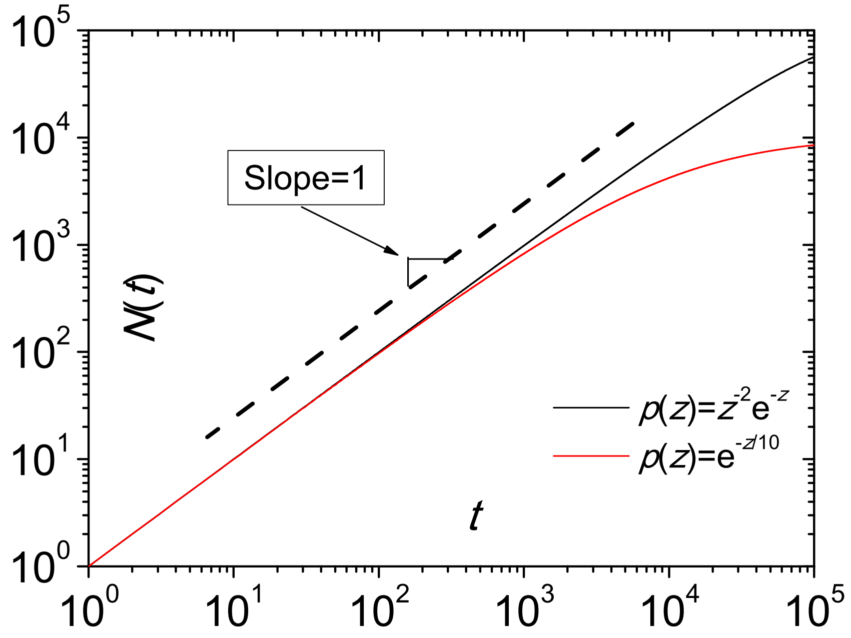

Figure S6: Comparison of the growing tendencies of $N(t)$ between the cases of power-law distribution with an exponential cutoff and the purely exponential distribution. The black and red solid lines denote the results generated by a power-law distribution with a very strong exponential cutoff ($p(z) \sim z^{-2}\exp(-z)$) and a purely exponential distribution ($p(z) \sim \exp(-z/10)$). The dash line is of slope 1 for eye guidance.
